# Pentraxin 3 in primary percutaneous coronary intervention for ST elevation myocardial infarction is associated with early irreversible myocardial damage: Kinetic profile, relationship to interleukin 6 and infarct size

**DOI:** 10.1177/2048872620923641

**Published:** 2020-05-14

**Authors:** Noreen Butt, LK Bache-Mathiesen, A Ushakova, JE Nordrehaug, SE Jensen, PS Munk, N Danchin, JL Dubois-Rande, HS Hansen, F Paganelli, P Le Corvoisier, H Firat, D Erlinge, D Atar, AI Larsen

**Affiliations:** 1 Department of Clinical Science, University of Bergen, Norway; 2 Department of Cardiology, Stavanger University Hospital, Norway; 3 Department of Research, Section of Biostatistics Stavanger, University Hospital, Norway; 4 Cardiology, Aalborg University Hospital, Denmark; 5 Department of Cardiology, Sørlandet Hospital, Norway; 6 Cardiology, Hôpital Européen Georges Pompidou, Université Paris Descartes, France; 7 Cardiology and Clinical Investigation Center, University Hospital Henri Mondor, France; 8 Odense University Hospital, Denmark; 9 Hospital Nord of Marseille, France; 10 Firalis SA, France; 11 Lund University, Sweden; 12 Department of Cardiology, Oslo University Hospital Ullevål and University of Oslo, Norway

**Keywords:** STEMI, primary percutaneous coronary intervention, pentraxin 3, interleukin 6, high-sensitive C-reactive protein, inflammation

## Abstract

**Background:**

The inflammatory marker long pentraxin 3 (PTX3) has been shown to be a strong predictor of 30-day and one-year mortality after acute myocardial infarction. The aim of this study was to evaluate the kinetic profile of PTX3 and its relationship with interleukin 6 (IL-6), high-sensitive C-reactive protein (hs-CRP) and infarct size.

**Methods:**

PTX3, IL-6 and hs-CRP were measured at predefined time points, at baseline (before percutaneous coronary intervention (PCI)), at 12 and 72 hours after PCI in 161 patients with first-time ST elevation myocardial infarction (STEMI).

**Results:**

PTX3 and IL-6 levels increased in *the early phase*, followed by a gradual decrease between 12 and 72 hours. There were statistically significant correlations between PTX3 and IL-6 in general, for all time points and for *changes* over time (0–72 hours). In a linear mixed model, PTX3 predicted IL-6 (*p* < 0.001). PTX3 is also correlated with hs-CRP in general, and at each time point post PCI, except at baseline. PTX3, IL-6 and hs-CRP were all significantly correlated with infarct size in general, and at the peak time point for maximum troponin I. In addition, there was a modest correlation between IL-6 levels at baseline and infarct size at 72 hours after PCI (*ρ* = 0.23, *p* = 0.006).

**Conclusions:**

PTX3 had a similar kinetic profile to IL-6, with an early increase and decline, and was statistically significantly correlated with markers of infarct size in STEMI patients post primary PCI. Baseline levels of IL-6 only predicted infarct size at 72 hours post PCI.

## Introduction

Despite timely reperfusion by primary percutaneous coronary intervention (pPCI) and optimal medical treatment in patients admitted with ST elevation myocardial infarction (STEMI), some patients develop large infarcts with adverse left ventricular remodelling. In addition to the reperfusion damage initiated by radical oxygen species, the extent of cardiac injury also depends on the level of inflammation and subsequent immune cell recruitment. An inflammatory phase disproportionately prolonged, of excessive magnitude, or insufficiently suppressed, can lead to sustained tissue damage and improper healing, promoting infarct expansion, adverse remodelling and chamber dilatation.^1^

The MITOCARE trial evaluated whether the administration of the mitochondrial permeability transition pore (mPTP) inhibitor TRO40303 prior to pPCI could reduce reperfusion injury.^2^ However, the trial failed to show a cardioprotective effect of the substance.^3^

The secretion of interleukin 6 (IL-6), a prototypical cytokine, which is the major determinant of the production of the acute-phase proteins, C-reactive protein (CRP; a short pentraxin), is increased in infarcted myocardium.^4^ Moreover, elevated levels of both IL-6 and CRP correlate with infarct size,^5^ and elevated levels of CRP relate to increased in-hospital mortality and a worse prognosis.^6–11^ The role of a relatively new biomarker in myocardial infarction, the long pentraxin 3 (PTX3), is less understood.^12^

Several cell types release PTX3 in response to inflammation,^13^ and PTX3 is a more specific biomarker of inflammation than CRP in atherosclerotic lesions. Circulating levels of PTX3 reflect the instability of coronary plaques and the *extent* of myocardial damage in acute myocardial infarction (AMI).^14^ It is well known that PTX3 is produced in response to inflammatory cytokines like IL-6.^15^ However, few studies have evaluated the kinetic profile and the possible prognostic significance of altered levels of PTX3 during STEMI. This may be of clinical interest since PTX3 can have protective anti-inflammatory properties. Moreover, it is not known whether the level of inflammation at admission before pPCI can predict infarct size.

The aims of the current study were as follows:

- To explore the *kinetic profile* of PTX3 and compare it with the kinetic profile of IL-6 and hs-CRP in first-time STEMI patients admitted for pPCI at predefined time points.
- To investigate if the levels of these biomarkers are associated with infarct size assessed by troponin I (TnI) and creatine kinase–myocardial band (CK-MB) at 72 hours post PCI.
- To evaluate whether hs-CRP and PTX3 can predict the level of IL-6 during the first 72 hours post PCI.

## Methods

### Patients

The MITOCARE study was a multicentre, randomized, double-blind, placebo-controlled trial (RCT) carried out in four European countries in the period October 2011–September 2013. Details of the study design have previously been reported.^3^ The study did not show any beneficial effect of the mPTP inhibitor TRO40303 in limiting the extent of reperfusion injury.

Briefly, the study population included patients > 18 years of age with a first-time STEMI, defined as nitrate-resistant chest pain ≥ 30 min, and new ST elevation at J-point in two contiguous leads with cut-off points: ≥ 0.2 mV in men or ≥ 0.15 mV in women in leads V2–V3 and/or ≥ 0.1 mV in other leads. Additional inclusion criteria were presentation within six hours of the onset of chest pain, clinical decision to treat with pPCI, occlusion of culprit artery with thrombolysis in myocardial infarction (TIMI) flow grade 0–1 at time of admission and before PCI. Patients were excluded if they had multi-vessel disease, experienced cardiac arrest with or without ventricular fibrillation, cardiogenic shock, stent thrombosis, a previous AMI, angina within 48 hours before infarction, previous coronary artery bypass graft, intravenous fibrinolysis within 72 hours prior to PCI, atrial fibrillation, had a pacemaker, concurrent inflammatory, infectious or malignant disease, or a biliary obstruction or hepatic insufficiency. The demographics of the study population are shown in Table 1.

**Table 1.** MITOCARE patient characteristics (*n* = 161).

Variable	Frequency
Age, years, median (interquartile range)	62 (53, 70)
Body mass index (BMI), kg/m^2^, median (interquartile range)	27.3 (25, 30)
Sex	
Male	135 (83.9%)
Female	26 (16.1%)
Diabetes	
Yes	12 (7.5%)
No	149 (92.5%)
Hypertension	
Yes	47 (29.2%)
No	114 (70.8%)
Smoking	
Yes	67 (46.2%)
No	78 (53.8%)
Stratum	
Anterior	64 (39.8%)
Posterior	97 (60.2%)
Killip class	
1	112 (70.4%)
2–5	47 (29.6%)

A signed informed consent to participate was obtained prior to any study-related procedure, or within 12/24 hours post-procedure if oral consent was provided beforehand (France/Norway). The study was in accordance with the Declaration of Helsinki and approved by the regional ethics committees.

### Blood sampling and analyses

Blood samples from 161 patients were analysed by the core lab FIRALIS (Huningue, France) to measure levels of PTX3, IL-6 and hs-CRP, and markers of myocardial necrosis CK-MB and TnI, before primary PCI and at 12- and 72-hours post PCI. PTX3 was quantified by use of a Human PTX3/TSG-14 Immunoassay Quantikine ELISA Kit. To reduce measurement errors, PTX3, IL-6 and hs-CRP were measured twice at the respective time points. These repeated measures showed high internal consistency (Cronbach’s α > 0.9, intraclass correlation coefficient > 0.9; online Appendix Table 1).

**Figure 1.**
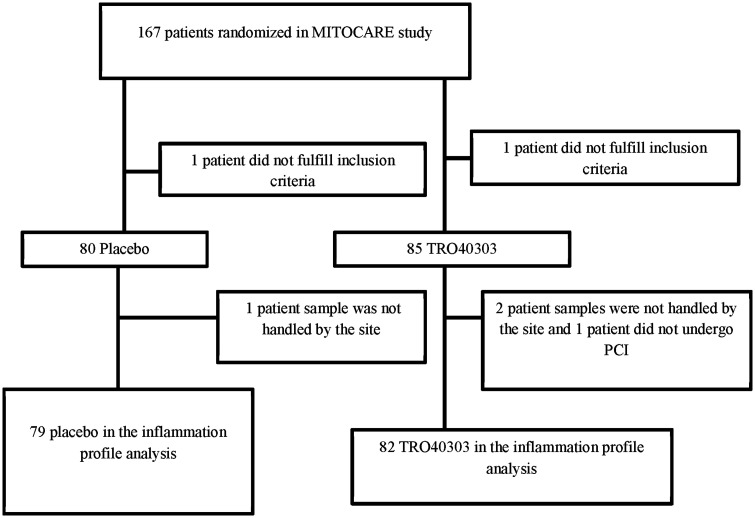
Patient flow chart.

Two patients did not undergo PCI and were removed from the dataset. The patient flow chart is shown in Figure 1.

We have previously shown that there were no statistically significant treatment effects of TRO40303 on the levels of PTX3, IL-6 or hs-CRP during the first 72 hours after primary PCI^16^ (online Appendix Table 2). Therefore, the kinetics of PTX3, IL-6 and hs-CRP were analysed in all 161 patients. TnI and CK-MB were analysed as markers of myocardial injury at the same time points as the inflammatory markers. Some patients had missing values for one or more time points (online Appendix Table 3). The detection method could not detect values below a certain threshold, and some values were removed due to measurement errors.

**Table 2.** Correlation between pentraxin 3, interleukin 6 and hs-CRP. Pearson correlations of log-transformed values in general (overall), between baseline levels (0 hours), 12 and 72 hours post PCI are calculated. For the change over time between these time points (0–12 hours, 0–72 hours and 12–72 hours), Spearman correlations are reported.

	Pentraxin 3	IL-6	Hs-CRP
Overall			
Pentraxin 3	1	0.36***	0.22***
IL-6	0.36***	1	0.40***
Hs-CRP	0.22***	0.40***	1
0 hours			
Pentraxin 3	1	0.23*	0.05
IL-6	0.23*	1	0.29***
Hs-CRP	0.05	0.29***	1
12 hours			
Pentraxin 3	1	0.20*	0.30**
IL-6	0.20*	1	0.41***
Hs-CRP	0.30**	0.41***	1
72 hours			
Pentraxin 3	1	0.47***	0.48***
IL-6	0.47***	1	0.53***
Hs-CRP	0.48***	0.53***	1
0–12 hours			
Pentraxin 3	1	0.18	0.33**
IL-6	0.18	1	0.31***
Hs-CRP	0.33**	0.31***	1
0–72 hours			
Pentraxin 3	1	0.33**	0.43***
IL-6	0.33**	1	0.38***
Hs-CRP	0.43***	0.38***	1
12–72 hours			
Pentraxin 3	1	0.02	0.11
IL-6	0.02	1	0.29**
Hs-CRP	0.11	0.29**	1

*p < 0.05.

**p < 0.01.

***p < 0.001.

**Table 3.** Spearman’s Rho (*ρ*) correlations between PTX3, IL-6, hs-CRP and markers of infarct size CK, CK-MB and TnI.

	PTX3	IL-6	Hs-CRP
Overall			
CK	0.31***	0.48***	0.21***
CK-MB	0.37***	0.49***	0.18***
TnI	0.36***	0.55***	0.38***
Peak time point (12 hours)†			
CK	0.24*	0.32***	0.33***
CK-MB	0.19*	0.26**	0.29***
TnI	0.26**	0.28***	0.35***
PTX3, IL-6 and hs-CRP at baseline, infarct size after 72 hours§			
CK	0.11	0.15	0.03
CK-MB	0.09	0.14	0.01
TnI	0.08	0.23**	–0.02

*p < 0.05.

**p < 0.01.

***p < 0.001.

†The time point with the maximum median values for infarct size, CK, CK-MB and TnI.

§Whether values of PTX3, IL-6 and hs-CRP at baseline are correlated with markers of infarct size 72 hours post PCI.

## Statistical analysis

We used the average value of two measurements taken at the same time for the correlation analysis for each biomarker. Pearson’s correlation analyses were performed between log-transformed values of PTX3, IL-6 and hs-CRP in general, and for each time point at 0, 12 and 72 hours after PCI. Spearman’s correlations (*ρ*) were analysed for changes in biomarkers over specific time periods (0–72 h, 0–12 h and 12–72 h). The correlation tests were repeated in the group of patients with TIMI flow grade 3 after PCI to adjust for TIMI flow if it was a potential confounder.

To assess whether there were relationships between infarct size and levels of PTX3, IL-6 and hs-CRP, Spearman’s correlations were evaluated between the acute-phase proteins and the markers of infarct size (CK, CK-MB and TnI) in general and at specific time points post PCI. Similarly, Spearman’s correlation analyses were employed to assess the association between the changes in levels of PTX3 and/or IL-6 and markers of infarct size.

To evaluate whether, and of what magnitude, the amount of IL-6 can be predicted by levels of PTX3, a linear mixed model was used. Mixed models are well suited to control for within-cluster dependencies between patients.^17^ Mixed models can also take into account dependencies between repeated measurements. Thus, the initial model included the random effects of (a) variability between individuals in repeated measures; (b) variability between individuals within the same centre; (c) variability between values at different time points within an individual; and (d) variability in the slope of PTX3 between individuals. Selection of random effects for the final model was determined by Akaike’s information criterion (AIC). The correlation matrix structure used in the model was compound symmetry.

The following effects were tested as potential confounders of the effect of PTX3 on IL-6: sex (male/female), age (years), time (0/12/72 hours), smoking (yes/no), hypertension (yes/no), diabetes (yes/no) and stratum (anterior/posterior). The model with the most accurate estimate of PTX3 was determined by the best AIC. The Kenward–Roger approximated *F-*test was used for estimation of *p*-values. Outlier influence was evaluated with Cook’s Distance. PTX3 and IL-6 were transformed by the natural logarithm. The same procedure was used to find out whether, and of what magnitude, the amount of log-transformed IL-6 could be predicted by levels of log-transformed hs-CRP.

A significance level of *α* = 0.05 was chosen for all models and tests. The Statistical Package for the Social Sciences (SPSS, Chicago, IL, USA) version 23 was used for correlations. R version 3.6, with the packages lme4 version 1.1-21, lmtest version 0.9-37, influence.ME version 0.9-9 and lmerTest version 3.1-1 were used for mixed model estimations and model checking. The package corrplot version 0.84 was used to create correlation plots.

## Results

### Kinetics

**Figure 2.**
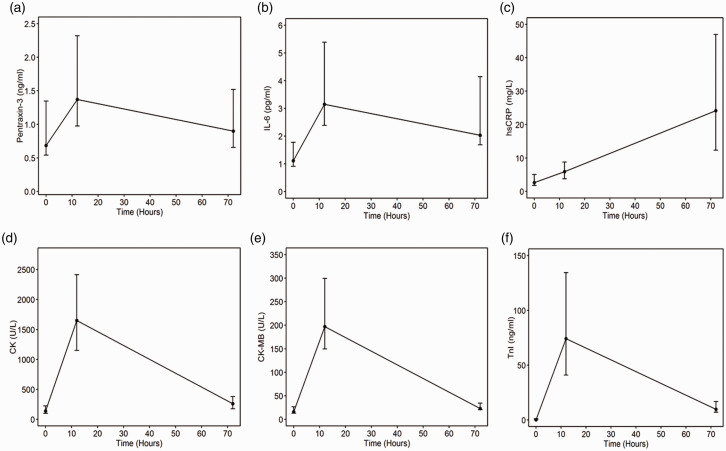
The kinetic profile of PTX3, IL-6, hsCRP, CK, CK-MB and TnI during the first 72 hours post PCI. Shown for levels of: (a) PTX3; (b) IL-6; (c) hsCRP; (d) CK; (e) CK-MB; (f) TnI. Round points denote the median level of the corresponding biomarker at the time measured; zero, 12 and 72 hours. Whiskers represent interquartile range. PTX3: pentraxin 3; IL-6: interleukin 6; hsCRP: high-sensitive C-reactive protein; CK: creatinine kinase; CK-MB: creatine kinase–myocardial band; TnI: troponin I.

In contrast to hs-CRP (Figure 2(c)), PTX3 and IL-6 levels increased in *the early phase* of first-time single-vessel STEMI and then gradually decreased between 12 and 72 hours after PCI (Figure 2(a) and (b)). The same pattern was seen for the markers of infarct size (Figure 2(d) to (f)).

### Correlation between pentraxins and IL-6

The correlations for all time points between PTX3, IL-6 and hs-CRP are reported in Table 2. PTX3 and IL-6 showed a weak but statistically significant correlation at each time point (*p* < 0.05; Table 2).

**Figure 3.**
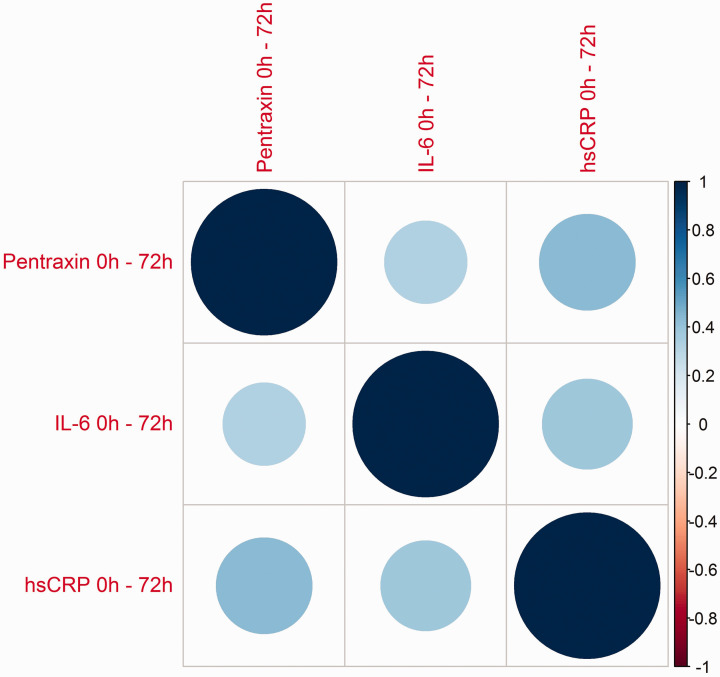
Correlation plot showing the magnitude and direction of correlations between changes in levels of PTX-3, IL-6 and hsCRP from baseline to 72 hours. Greater size and colour intensity of circles indicates a higher correlation between markers in the correlation plot. PTX3: pentraxin 3; IL-6: interleukin 6; hsCRP: high-sensitive C-reactive protein.

This result was consistent with correlations between *changes* of PTX3 and IL-6 from zero to 72 hours. (*p* ≤ 0.001; Table 2, Figure 3). Hs-CRP was positively correlated with IL-6 at each time point and with PTX3 at 12 and 72 hours (*p* < 0.001). However, there was no significant association between hs-CRP and PTX3 at zero hours (Table 2). There was a highly statistically significant correlation between the changes in levels of PTX3 and hs-CRP from 0 to 12 and 0 to 72 hours and for IL-6 and hs-CRP at the same time intervals (*p* < 0.001; Table 2, Figure 3). However, the correlation between the changes in levels of PTX3 and IL-6 was only statistically significant at 0–72 hours (*p* < 0.001; Table 2, Figure 3). The correlation analyses performed in patients with TIMI 3 flow only (*n* = 129) did not substantially change the effect size or the levels of statistical significance for any of the correlations for the whole cohort.

### Infarct size

PTX3, IL-6 and hs-CRP were, to a varying degree, significantly correlated with infarct size in general, and at the peak time point of infarct size (Table 3).

**Figure 4.**
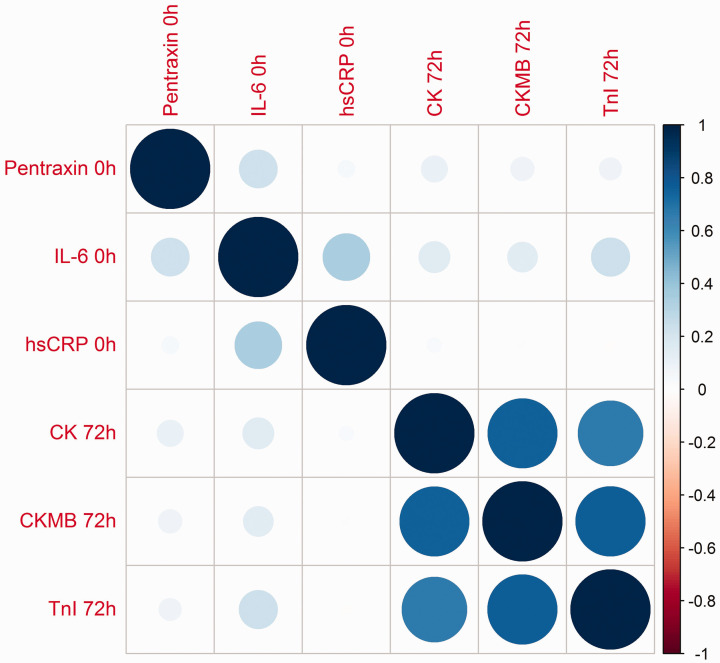
Correlation plot showing the magnitude and direction of correlations between PTX3, IL-6 and hsCRP levels at baseline and markers of infarct size, TnI, CK and CK-MB levels, 72 hours post PCI. Greater size and colour intensity of circles indicates a higher correlation between markers in the correlation plot. PTX3: pentraxin 3; IL-6: interleukin 6; hsCRP: high-sensitive C-reactive protein; CK: creatinine kinase; CK-MB: creatinine kinase–myocardial band; TnI: troponin I; PCI: percutaneous coronary intervention.

**Figure 5.**
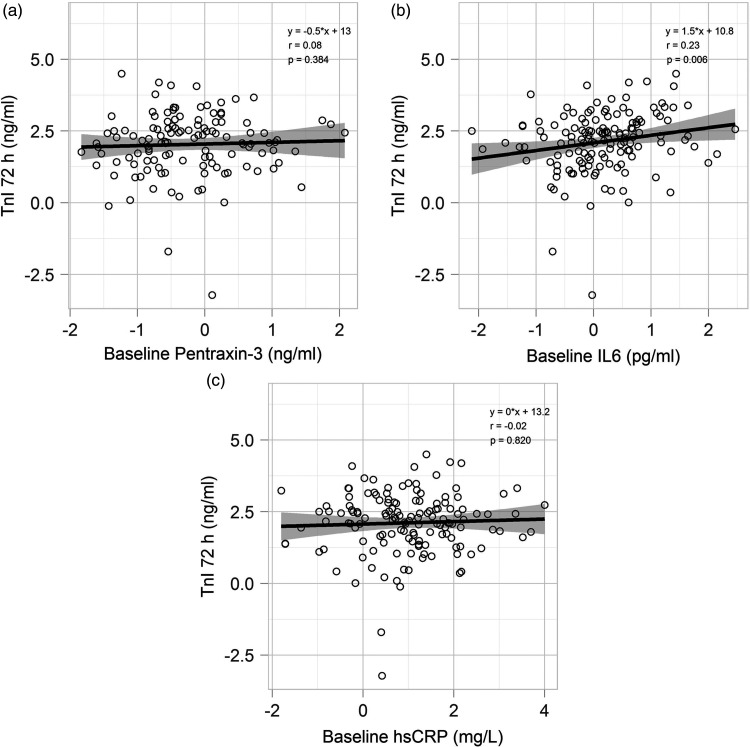
Scatterplots showing the relationship between (a) PTX-3 levels at baseline and TnI levels 72 hours post PCI; (b) baseline IL-6 and TnI levels 72 hours post PCI; (c) baseline hsCRP and TnI levels 72 hour post PCI. PTX3: pentraxin 3; IL-6: interleukin 6; hsCRP: high-sensitive C-reactive protein; TnI: troponin I; PCI: percutaneous coronary intervention.

IL-6 levels *at baseline* were statistically significantly, but only modestly, correlated with markers of myocardial injury (TnI) at 72 hours after PCI (*ρ* = 0.232, *p* = 0.006; Table 3, Figures 4 and 5(c)); otherwise, neither PTX3 nor hs-CRP levels *at baseline* were related to TnI or CKMB at 72 hours post PCI (Table 3, Figures 4 and 5(a), (b)).

### Prediction of IL-6

Log-transformed values of hs-CRP and PTX3 were both statistically significant predictors of log-transformed IL-6 levels in the linear mixed model (hs-CRP: *β* = 0.28, *p* < 0.001; PTX3: *β* = 0.25, *p* ≤ 0.001; Table 4).

**Table 4.** Coefficient estimate *β* and Kenward–Roger *p*-values estimated from a linear mixed model to determine whether levels of pentraxin 3 can predict levels of interleukin 6, and, likewise, for whether hs-CRP can predict levels of IL-6.

Model with pentraxin 3			Model with hs-CRP		
Fixed effects	Estimates (95% CI)	*P*-value†	Fixed effects	Estimates (95% CI)	*P*-value†
Intercept	0.02 (–0.55, 0.6)	0.932	Intercept	–0.51 (–0.98, –0.04)	0.034
log pentraxin3*	0.25 (0.14, 0.36)	<0.001	log hs-CRP*	0.28 (0.21, 0.35)	<0.001
Age (years)	0.01 (0.00, 0.02)	0.115	Age (years)	0.01 (0.00, 0.01)	0.070
Sex			Sex		
Female	0		Female		
Male	0.21 (–0.06, 0.48)	0.124	Male	0.13 (–0.10, 0.36)	0.275
Time			Time		
0	0		0		
12	0.91 (0.73, 1.09)	<0.001	12	0.8 (0.64, 0.95)	<0.001
72	0.61 (0.44, 0.79)	<0.001	72	–0.04 (–0.25, 0.16)	0.674

*Interleukin 6, pentraxin 3 and hs-CRP were transformed by the natural logarithm for this model.

†Based on Kenward–Roger *F*-test.

## Discussion

In the current study, levels of PTX3 increased during the first 12 hours, followed by a decrease towards 72 hours post PCI. This is in contrast to the prolonged increase known for hs-CRP. In addition, PTX3 was associated with infarct size during the first 12 hours of STEMI. This indicates that PTX3 is associated with irreversible myocardial damage, supporting the prognostic significance of admission and peak PTX3. Moreover, IL-6 at baseline was a modest but statistically significant predictor of infarct size at 72 hours.

### 1) Kinetic profile of PTX3 compared with the kinetic profile of IL-6 and hs-CRP in first-time single-vessel STEMI

Pentraxins are essential components of the innate immunity response and are divided into short pentraxins such as CRP, mainly produced by liver cells in response to IL-6 and long PTX3.^15^ Whereas both CRP and PTX3 are well-known biomarkers of inflammation and predict prognosis in cardiovascular disease,^18,19^ the long PTX3 differs from CRP, in gene organization, chromosomal localization, cellular sources and in the ability to induce stimuli and recognize ligands.^20^

In contrast to hs-CRP, which seems to increase beyond 72 hours, the current study showed that levels of PTX3 (and IL-6) increased during the first 12 hours and then decreased towards 72 hours post pPCI in first-time STEMI patients. This is in accordance with previous research, indicating that plasma levels of PTX3 seem to be *normalized* within 48 hours after the onset of symptoms.^21^ The fast increase is depending on the release of PTX3 from granules in neutrophil leucocytes, which occur within six hours after plaque rupture in AMI. The subsequent gradual decline after the peak of 12 hours is, on the other hand, mostly due to the short half-life of the circulating neutrophil granulocytes.^12,21,22^ On the contrary, CRP is produced in the liver cells stimulated by IL-6. The kinetic profile previously described for CRP is in accordance with the current study in which the actual measurements were done at zero, 12 and 72 hours.

#### Mechanisms of action of PTX3

After reperfusion injury, the lack of PTX3 has been shown to be associated with increased myocardial damage, characterized by no-reflow area, increased neutrophil infiltration, increased number of apoptotic cells and decreased number of capillaries. In addition, C3 complement component has been shown to increase focally, being related to the area of damaged myocardium. In PTX3 knock-out mice, the administration of exogenous PTX3 reduces complement C3 deposition, further indicating cardioprotective effects of PTX3 by the modulation of the complement cascade.^23^

The released PTX3 also binds to activated circulating platelets, resulting in the *reduction* of their pro-inflammatory and prothrombotic effects,^21^ supporting the view that PTX3 also have atheroprotective effects.^24^ The physiological properties and role of PTX3 are not fully understood, but current evidence support that PTX3 might have both pro-inflammatory and anti-inflammatory effects depending on the context of the action.^13^

### 2) Prognostic importance of PTX3, IL-6 and hs-CRP

Despite these potentially beneficial effects of PTX3,^25–27^ elevated levels are associated with the magnitude of myocardial damage. In addition, high PTX3 levels are a predictor for increased morbidity and mortality in STEMI patients undergoing pPCI.^28–30^ A positive correlation between levels of PTX3, CRP and metalloproteinase-9, also underline the importance of PTX3 on prognosis in this population.^31,32^ PTX3, IL-6 and CRP all have prognostic value in AMI. Ammirati et al. proposed a risk index that combines IL-6 with IL-10 to predict outcome in STEMI patients.^33^ The effect on prognosis is partly related to an effect on remodelling.

#### Myocardial necrosis and inflammation; the role of pentraxins’ relationship to prognosis

It is well recognized that elevated levels of circulating IL-6 in acute coronary syndromes are of prognostic value.^34–36^ IL-6 binds to plasma membrane receptor complexes in the heart, activating two major signalling cascades, SHP2/ERK and STAT pathways that are important for the remodelling process in the myocardium.^37^

In accordance with this, a relationship between IL-6 and the end-diastolic diameter of the left ventricle at long-term follow-up has been demonstrated.^38^ In addition, both circulating levels of IL-6 and CRP have shown to be associated with the extent of myocardial necrosis.^39^

In contrast to the current findings, an *experimental* model has demonstrated that low levels of PTX3 were associated with high levels of IL-6 and extended myocardial damage. This was related to the ischemia-reperfusion injury, in that PTX3 deficient mice develop increased myocardial damage, characterized by no-reflow area, increased neutrophil infiltration apoptotic cells and decreased number of capillaries.^23^ The coronary circulation is the main source of PTX3 in heart failure patients with normal ejection fraction, and levels of PTX3 correlate with the degree of left ventricular diastolic dysfunction.^40^ This identification of myocardial tissue as a main source for circulating levels of PTX3 indicates that PTX3 is an early marker of irreversible myocyte injury in ischemic cardiomyopathy. Systemic pre-PCI levels of PTX3 have been shown to be associated with high-risk plaque components and impaired post-PCI myocardial perfusion.^30^ It has, therefore, been speculated that PTX3 might act as a potential novel biomarker of myocardial infarction.

Accordingly, in the current study we found that PTX3, IL-6 and hs-CRP are correlated with markers of myocardial necrosis during the first 12 hours of myocardial infarction (Table 4). Moreover, there was a statistically significant correlation between PTX3 and IL-6 at all time points, but no statistically sigificantly correlation between PTX3 and hs-CRP at baseline (Table 2) hs-CRP. This relationship is further confirmed in the finding that both PTX3 and hs-CRP could predict IL-6 response (Tables 3 and 4).

In the current study, we found that IL-6 at baseline was a modest but statistically significant predictor of infarct size at 72 hours. This may indicate that the level of inflammation at baseline is an important factor for infarct size and subsequent left ventricular function and prognosis. Thus, PTX3, IL-6 and CRP all have prognostic value in AMI.

#### Study strengths and limitations

The weakness of the study is the few time points for analysis. In addition, the time from symptom debut to admission is often difficult to assess and confirm. On the other hand, the strength of the current study is the prospective design with blood samples drawn at specific pre-defined time points. The blood samples were immediately processed and stored. The population is relatively homogenous with first-time STEMI, with the occlusion of one of the major coronary branches. The inclusion criteria excluded patients with symptoms beyond six hours. The study was a multicentre RCT, with all analyses performed at one core lab.

## Conclusion

In first-time STEMI patients post primary PCI, PTX3 and IL-6 had a similar kinetic profile with an early increase and decline in contrast to the pattern seen for hs-CRP. In addition, levels of PTX3 were statistically significantly correlated with markers of infarct size. Finally, the infarct size at 72 hours post PCI was predicted only by baseline levels of IL-6 and not baseline levels of PTX3.
